# The impacts of surgery of the primary cancer and radiotherapy on the survival of patients with metastatic rectal cancer

**DOI:** 10.18632/oncotarget.19157

**Published:** 2017-07-11

**Authors:** Duo Tong, Fei Liu, Wenhua Li, Wen Zhang

**Affiliations:** ^1^ Department of Medical Oncology, Fudan University Shanghai Cancer Center, Shanghai 200032, China; ^2^ Department of Gynecological Oncology, Fudan University Shanghai Cancer Center, Shanghai 200032, China; ^3^ Department of Oncology, Shanghai Medical College, Fudan University, Shanghai 200032, China

**Keywords:** rectal cancer, surgery, radiotherapy, SEER, survival

## Abstract

The role of surgery of the primary cancer and radiation in metastatic colorectal cancer (mCRC) is still controversial currently, and evidence implied that colon cancer (CC) and rectal cancer (RC) should be treated with difference. Hence we focused on metastatic rectal cancer (mRC) solely to compare the cancer cause-specific survival (CSS) of patients receiving varied treatments of the primary cancer: no treatment, surgery only, radiation only, and surgery plus radiation, based on the records of the Surveillance, Epidemiology, and End Results (SEER) database. A total of 8669 patients were included. Results demonstrated that the 2-year CSS was 28.1% for no treatment group, 30.7% for only radiation group, 50.2% for only surgery group, and 66.5% for surgery plus radiation group, reaching statistical difference (*P* < 0.001). Furthermore, the CSSs of mRC patients in the surgery group were similar regardless of resection ranges (*P* = 0.44). Besides, we analyzed the prognostic factors for mRC and found carcinoembryonic antigen (CEA) level, metastasis (M) stage, Tumor (T) stage, tumor size, differentiate grade, age and marital status should be taken into consideration when estimating the prognosis. Particularly, patients with normal CEA level or M1a stage showed a significant survival advantage. Overall, present study suggested that surgery of the primary cancer and radiation might help to improve the survival of mRC patients, especially when both treatments were conducted. Our results may assist clinicians to make better treatment strategy for patients with mRC.

## INTRODUCTION

Colorectal cancer (CRC) is one of the most frequently diagnosed cancers worldwide, ranking the second leading cause of cancer-related death in the United States [1, 2]. In China, CRC is also among the five most common cancers and is the fifth leading cause of cancer-related death in both sexes [3]. Over half of CRC patients will develop metastases [4–6], and the 5-year relative survival rate of metastatic colorectal cancer (mCRC) is merely 10% [7]. So it is urgent to improve the survival of mCRC patients. Although commonly referred as CRC, colon cancer (CC) and rectal cancer (RC) differ in embryological origin, anatomical features and molecular pathologic traits, leading to distinct clinical characteristics [8, 9]. Considering the differences between CC and RC, and the fact that RC is more prevalent in China, we focus on metastatic rectal cancer (mRC) solely in this study [10].

For decades, great progress has been achieved in the field of the treatment of mRC. The utilization of several cytotoxic drugs [11–13]and targeted drugs [14, 15] has greatly improved the prognosis of patients. However, the 5-year relative survival rate of mRC still remains unsatisfactory. Chemotherapy is currently the primary treatment of mRC, while previous studies in RC implied that surgery of the primary cancer or radiotherapy might also play a positive role in the control of metastatic disease [16, 17]. The NCCN guidelines stratify mRC patients based on their status of metastases and include surgery and/or radiotherapy as potential treatment options for different groups. Recent studies found that varied treatment options of the primary cancer might exert different effects on the survival of gastrointestinal cancer patients [18, 19]. However, similar study in a large population focusing on mRC solely is currently scarce.

Moreover, traditional TNM (T, tumor; N, lymph node; M, metastasis) staging system is commonly used to guide the treatment and predict the prognosis of patients with RC. While for patients with mRC, some show worse survival than the others in spite of the same TNM stage. So extra prognostic factors may exist to help predict the outcome and tailor the treatment more precisely for mRC patients. According to the reports, some demographic and clinicopathological factors have already been found to be associated with the survival of gastrointestinal cancer patients [20–23]. Here we also intended to explore potential prognostic factors for mRC patients within the scope of demographic and clinicopathological factors.

So we conducted this study in mRC to investigate the relationship between cancer cause-specific survival (CSS) and the treatment options of primary cancer, as well as to find the demographic and clinicopathological prognostic factors, based on the Surveillance, Epidemiology, and End Results (SEER) database.

## RESULTS

### Patient baseline characteristics

We identified 8669 mRC patients in total for analysis from January 2004 to December 2013 based on the SEER database. Patients’ baseline demographics and clinicopathological characteristics were displayed in Table 1. 5385(62.1%) males and 3284(37.9%) females were included, mainly Caucasian (79%). The average age of patients was 61.2 years.

**Table 1:** Baseline demographic and clinicopathological characteristics of mRC patients in the SEER database (2004–2013)

Characteristics	*N* (%) (Total *N* = 8669)
**Gender**	
Male	5385 (62.1)
Female	3284 (37.9)
**Age at diagnosis (year)**	
< 60	4059 (46.8)
≥ 60	4610 (53.2)
Mean ± SD	61.2 ± 13.7 (years)
**Year of diagnosis**	
2004–2009	4973 (57.4)
2010–2013	3696 (42.6)
**Ethnicity**	
Caucasian	6852 (79.0)
Non-Caucasian	1794 (20.7)
Unknown	23 (0.3)
**Marital status**	
Married	4322 (49.9)
Unmarried	3987 (46)
Unknown	360 (4.1)
**Histology**	
Adenocarcinoma	8136 (93.9)
Mucinous adenocarcinoma	385 (4.4)
Signet ring cell carcinoma	148 (1.7)
**Grade**	
Well	392 (4.5)
Moderate	4861 (56.1)
Poor	1513 (17.5)
Undifferentiated	109 (1.3)
Unknown	1794 (20.7)
***T* stage**	
T1	1127 (13.0)
T2	300 (3.5)
T3	3353 (38.7)
T4	1488 (17.2)
Tx	2401 (27.7)
***N* stage**	
N0	2887 (33.3)
N1	2677 (30.9)
N2	1582 (18.2)
Nx	1523 (17.6)
**M status**	
M1a	2176 (25.1)
M1b	6208 (72.6)
M1x	286 (3.3)
**Tumor size**	
0–4 cm	1686 (19.4)
4–8 cm	2567 (29.5)
> 8 cm	141 (1.6)
Unknown	4305 (49.5)
**CEA**	
Normal	977 (11.3)
Elevated	4927 (56.8)
Unknown	2765 (31.9)
**Surgery**	
No	5382 (62.1)
Yes	3247 (37.4)
1.Local tumor excision	356 (4.0)
2.Partial proctectomy	2025 (23.4)
3.Total proctectomy or proctocolectomy	678 (7.8)
4.Total proctectomy with an en bloc resection of other organs	100 (1.2)
5.Unknown resection range	88 (1.0)
Unknown	40 (0.5)
**Radiation**	
No	4874 (56.2)
Yes	3662 (42.2)
1.Beam radiation	3557 (41.0)
2.Radioactive implants	10 (0.1)
3.Combination of beam with implants or isotopes	6 (0.06)
4. Radioisotopes	4 (0.04)
5.Radiation, NOS	85 (1.0)
Unknown	133 (1.5)

Abbreviations: mRC, metastatic rectal cancer; *N*, number of patients; SD, standard deviation; *T*, tumor; *N* stage, lymph node stage; M, metastasis; M1x, metastasis without exact M stage; CEA, carcinoembryonic antigen; NOS, not otherwise specified.

### Analysis of the impacts of demographic and clinicopathologic characteristics on mRC patients’ CSS

As summarized in Table 2, no surgery of primary cancer (*P* < 0.001), no radiation (*P* < 0.001), M1b stage (*P* < 0.001), elevated CEA level (*P* < 0.001), older age (*P* < 0.001), unmarried status (*P* < 0.001), signet ring cell carcinoma (*P* < 0.001), undifferentiated grade (*P* < 0.001), larger tumor size (*P* < 0.001) and T1/4 stage (*P* < 0.001) were found to be risk factors for mRC patients’ survival by univariate log-rank test. All these factors were then identified as independent prognostic factors by multivariate Cox regression. Notably, the 2-year CSSs for T2 stage and T3 stage were 55.5% and 52.4% respectively, while the rates were 37.6% and 33.0% for T1 stage and T4 stage (*P* < 0.001) (Table 2 and Figure 5), suggesting the survival of patients with T2/T3 stage was better than that of patients with T1/T4 stage in mRC. Among the prognostic factors identified, surgery of the primary cancer and radiation were included, which we planed to study further.

**Table 2:** Analysis of the effects of demographic and clinicopathological characteristics on cancer cause-specific survival of mRC patients in the SEER database

Variable	2-year CSS	Univariate analysis		Multivariate analysis	
		Log rank χ2	*P* ^*a*^	HR (95% CI)	*P* ^*b*^
**Gender**		2.488	0.115		
Female	42.2%			0.899 (0.770–1.049)	0.746
Male	39.6%			Reference	
**Age at diagnosis**		230.1	**< 0.001**		
< 60	49.6%			0.682 (0.615–0.757)	**< 0.001**
≥ 60	33.3%			Reference	
**Year of diagnosis**		23.7	**< 0.001**		
2004–2009	39.7%			1.117 (0.581–2.149)	0.214
2010–2013	45.1%			Reference	
**Ethnicity**		5.057	**0.025**		
Caucasian	41.9%			1.025 (0.865–1.214)	0.251
Non-Caucasian	38.7%			Reference	
**Marital status**		116.0	**< 0.001**		
Married	46.2%			0.849 (0.763–0.944)	**0.003**
Unmarried	35.2%			Reference	
**Histology**		78.9	**< 0.001**		
Adenocarcinoma	41.9%			0.550 (0.379–0.797)	**0.002**
Mucinous	39.1%			0.620 (0.404–0.950)	**0.028**
Signet ring cell	10.4%			Reference	
**Grade**		128.1	**< 0.001**		
Well	45.9%			0.527 (0.332–0.834)	**0.006**
Moderate	47.4%			0.475 (0.320–0.706)	**< 0.001**
Poor	31.2%			0.759 (0.506–1.139)	0.183
Undifferentiated	27.7%			Reference	
***T* stage**		198.5	**< 0.001**		
T1	37.6%			0.703 (0.713–1.068)	0.187
T2	55.5%			0.443 (0.464–0.820)	**0.001**
T3	52.4%			0.657 (0.591–0.762)	**< 0.001**
T4	33.0%			Reference	
***N* stage**		20.9	**< 0.001**		
N0	41.0%			1.049 (0.900–1.222)	0.541
N1	46.7%			1.026 (0.901–1.168)	0.700
N2	47.3%			Reference	
***M* stage**		195.6	**< 0.001**		
M1a	55.8%			0.708 (0.607–0.826)	**< 0.001**
M1b	37.0%			Reference	
**Tumor size**		49.31	**< 0.001**		
0–4 cm	50.7%			0.694 (0.588–0.817)	**< 0.001**
4–8 cm	46.8%			1.005 (0.867–1.164)	0.951
> 8 cm	36.6%			Reference	
**CEA**		124.7	**< 0.001**		
Normal	56.4%			0.674 (0.589–0.771)	**< 0.001**
Elevated	38.4%			Reference	
**Surgery**		804.1	**< 0.001**		
No	29.3%			2.331 (2.062–2.635)	**< 0.001**
Yes	58.7%			Reference	
**Radiation**		202.1	**< 0.001**		
No	36.0%			1.294 (1.162–1.440)	**< 0.001**
Yes	47.4%			Reference	
**Surgery + Radiation**		923.9	**< 0.001**		
No + No	28.1%			3.097 (2.612–3.673)	**< 0.001**
No +Yes	30.7%			2.56 0(2.181–3.005)	**< 0.001**
Yes + No	50.2%			1.41 (1.235–1.610)	**< 0.001**
Yes +Yes	66.5%			Reference	

*P^a^*: *P* value for comparisons between groups by log-rank test.

*P^b^*: *P* value for comparisons between groups by multivariable Cox regression analysis adjusting for covariates.

Abbreviations: mRC, metastatic rectal cancer; CSS, cancer cause-specific survival; HR, hazard ratio; CI, confidence interval; T, tumor; N, lymph node; M, metastasis; CEA, carcinoembryonic antigen.

**Figure 1:**
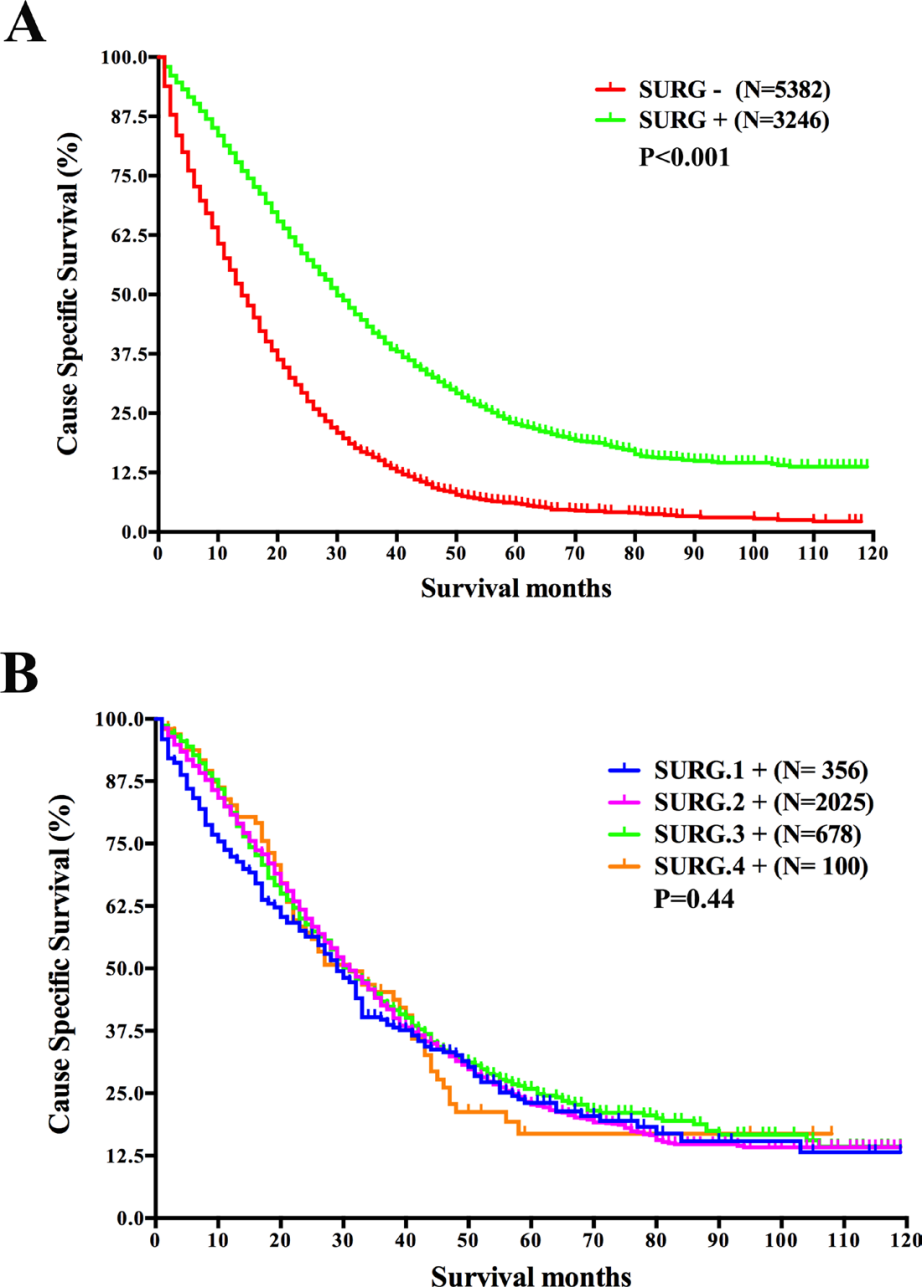
Survival analysis based on the status of surgery of the primary cancer and survival analysis based on surgical resection range in mRC (**A**) Survival analysis based on the status of surgery of the primary cancer. (**B**) Survival analysis based on surgical resection range in patients with surgery of the primary cancer. Abbreviations: *N*, number of patients; SURG−, without surgery of the primary cancer; SURG+, with surgery of the primary cancer; SURG.1+, the excision of local tumor; SURG.2+, partial proctectomy; SURG.3+, total proctectomy or total proctocolectomy; SURG.4+, total proctectomy or proctocolectomy with an en bloc resection of other organs, including pelvic exenteration.

**Figure 2:**
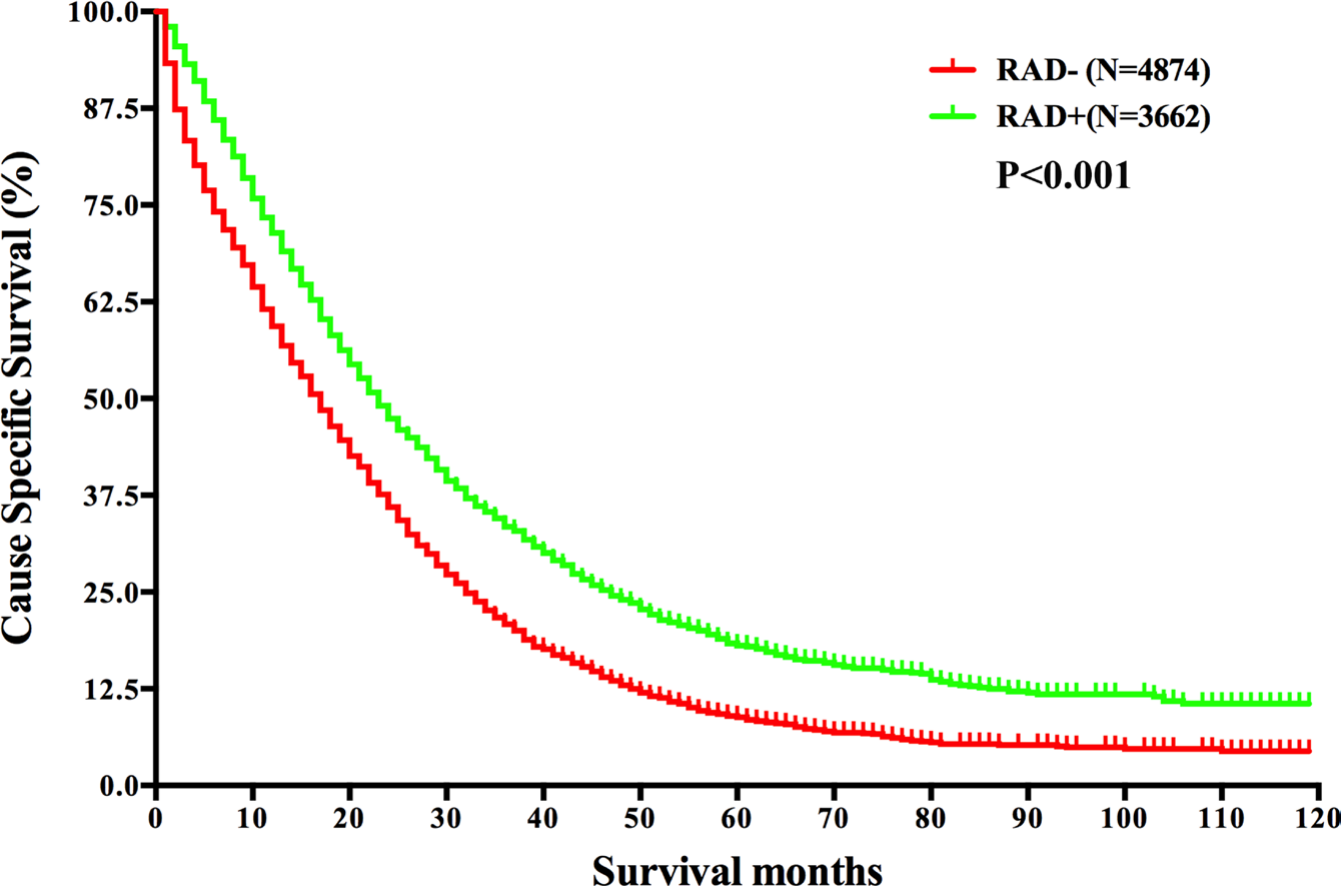
Survival analysis based on the status of radiation in mRC Abbreviations: *N*, number of patients; RAD−, without radiation; RAD+, with radiation.

**Figure 3:**
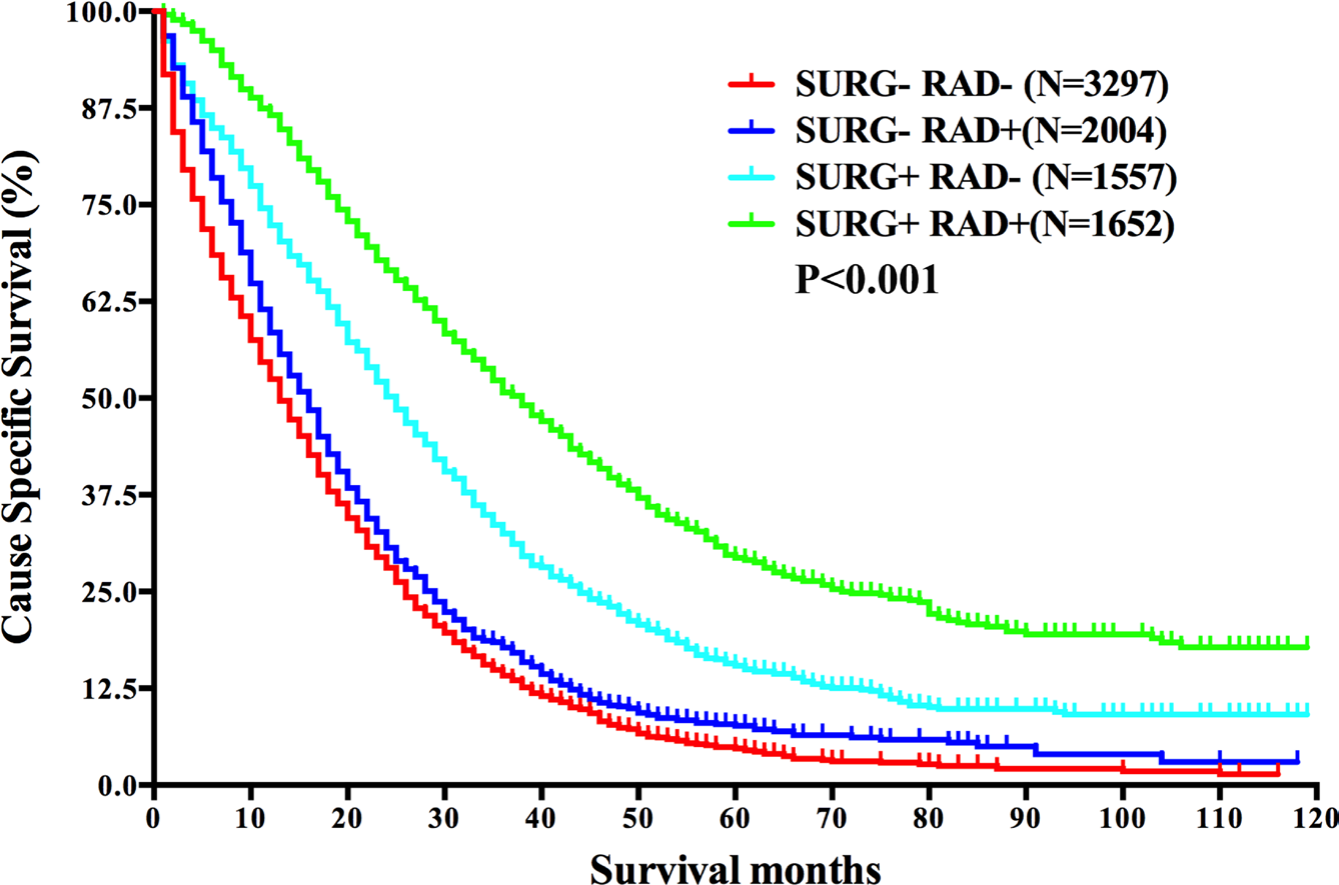
Survival analysis based on the status of both surgery and radiation in mRC Abbreviations: *N*, number of patients; SURG−, without surgery of the primary cancer; SURG+, with surgery of the primary cancer; RAD−, without radiation; RAD+, with radiation.

**Figure 4:**
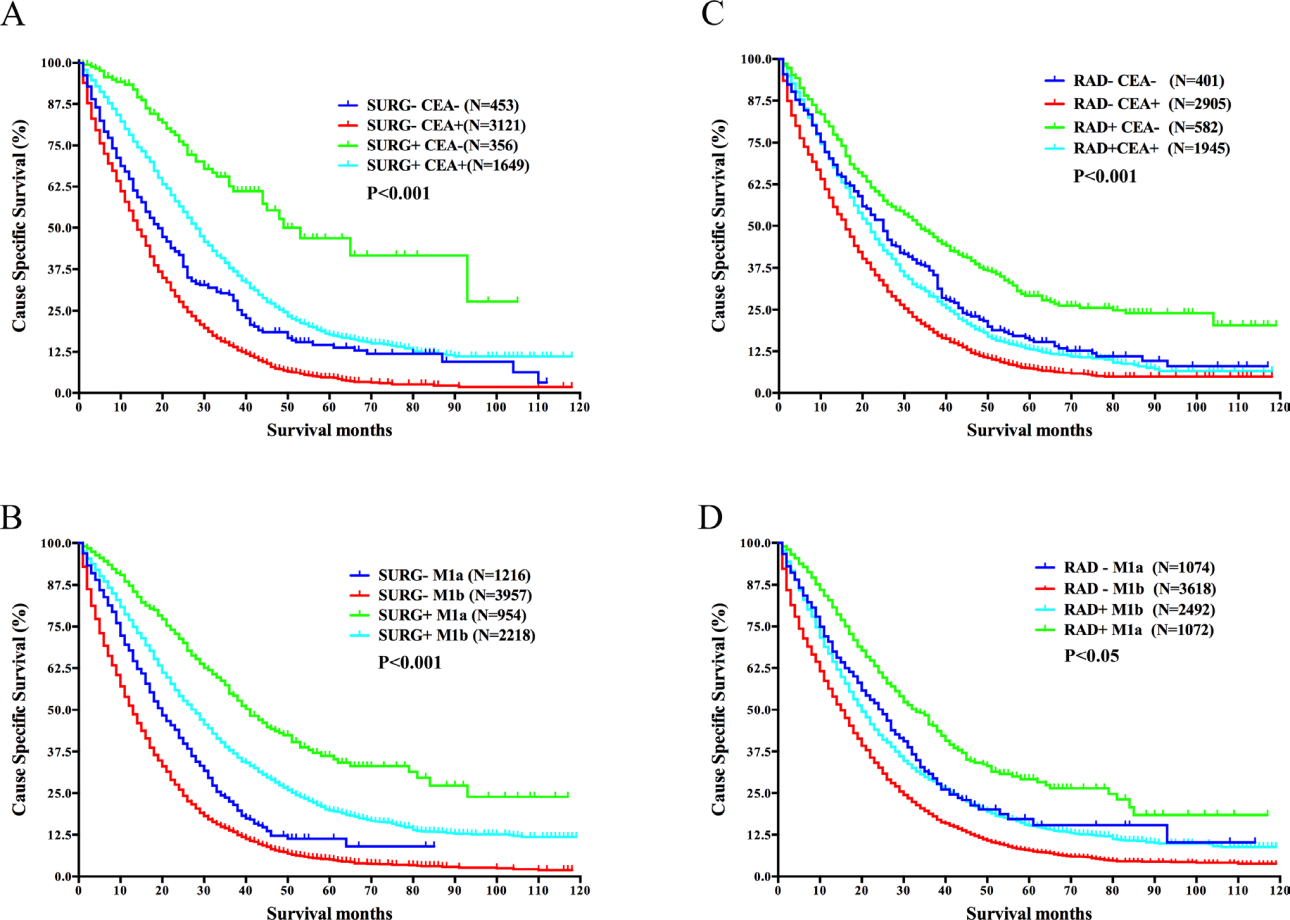
Survival analysis in different subgroups divided by surgery of the primary cancer or radiation and CEA level or *M* stage in mRC (**A**) Survival differences between subgroups divided by surgery and CEA level. (**B**) Survival differences between subgroups divided by surgery and M stage. (**C**) Survival differences between subgroups divided by radiation and CEA level. (**D**) Survival differences between subgroups divided by radiation and *M* stage. Abbreviations: *N*, number of patients; SURG−, without surgery of the primary cancer; SURG+, with surgery of the primary cancer; RAD−, without radiation; RAD+, with radiation; CEA−, with normal CEA level; CEA+, with elevated CEA level; M1a, M1a stage, metastasis confined to one organ/site; M1b, M1b stage, metastases to more than one organs/sites or peritoneum.

**Figure 5:**
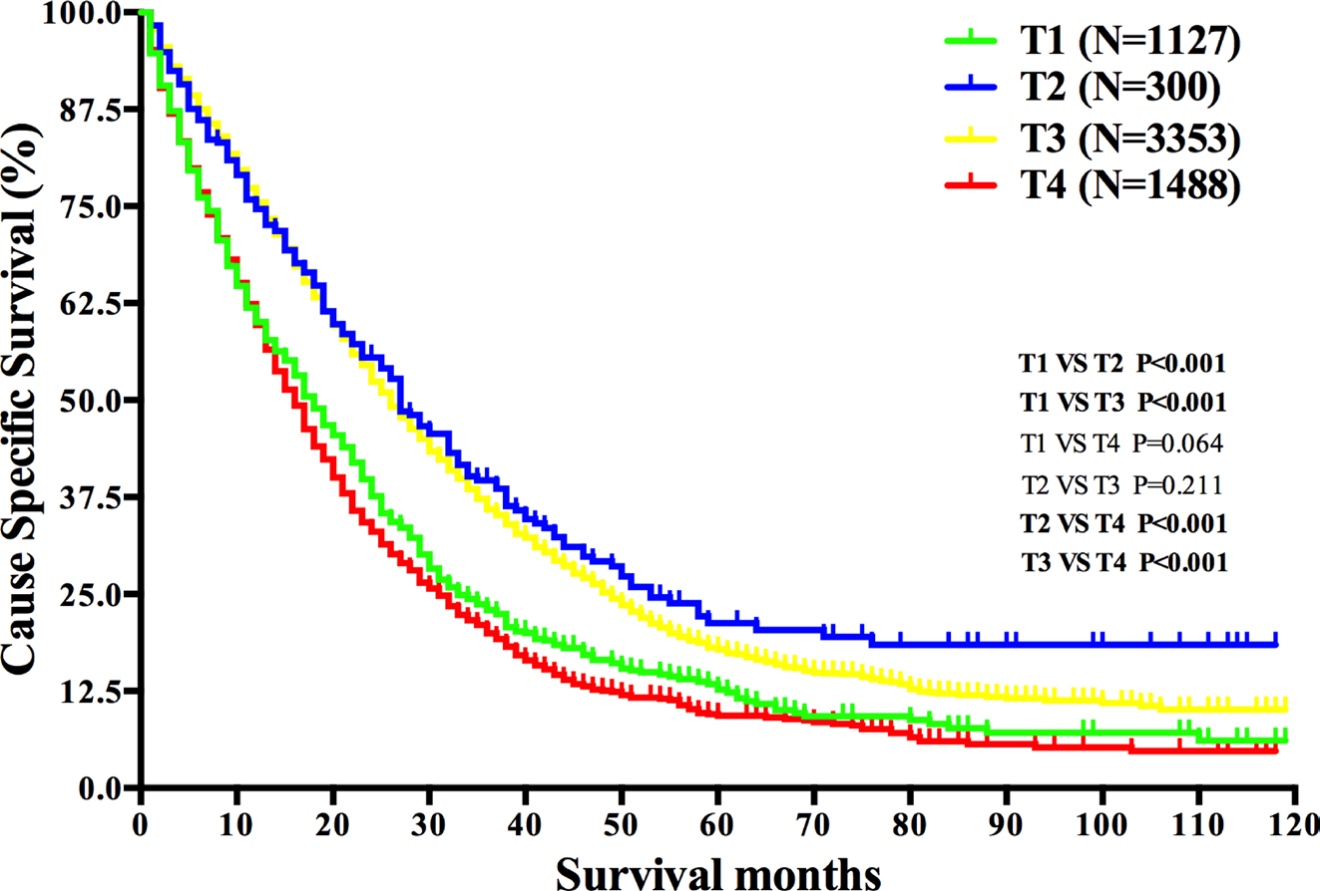
Survival analysis based on *T* stage in mRC Abbreviations: *N*, number of patients; *T*, tumor.

### Analysis of the impacts of surgery of the primary cancer on mRC patients’ CSS

In the cohort of analysis, among the 8629 (99.5%) patients with known information of surgery, 3247(37.6%) patients have performed surgery of the primary cancer. The 2-year CSS was 58.7% for patients who performed the surgery, while the rate was merely 29.3% for those who didn’t, reaching statistical significance by both univariate log-rank test and multivariate Cox regression analyses (*P* < 0.001) (Figure 1A and Table 2). We then analyzed the demographic and tumor characteristics in the two groups. Results demonstrated that patients in the surgery group were younger (*P* < 0.001), more married (*P* < 0.001), more with T3 stage (*P* < 0.001), more with smaller tumor size (*P* < 0.001), more with normal CEA level (*P* < 0.001) and more treated with radiotherapy (*P* < 0.001). The result implied that patients in the surgery group possessed more favorable survival factors.

As records in the SEER database also include the resection range of surgery, we further studied the prognostic differences within the surgery group according to the resection range. 3159 patients with known information of resection range were included for this analysis and they were divided into four groups based on the record. The result indicated that resection range did not affect the CSS of mRC patients (*P* = 0.44) (Figure 1B). It seemed that as long as the primary tumors were removed, mRC patients’ prognoses would be similar regardless of varied resection ranges.

### Analysis of the impacts of radiation on mRC patients’ CSS

Of the whole cohort, 8536 (98.5%) mRC patients owned known information of radiotherapy and among them 3662 (42.9%) have received radiation. The 2-year CSS was 47.4% for patients who received radiation, compared with 36.0% for those who did not, reaching statistical difference by both univariate and multivariate analyses (*P* < 0.001) (Figure 2 and Table 2).

Considering the combination of surgery of the primary cancer and radiation may achieve better survival as reported in local advanced disease [19], we then conducted survival analysis according to varied treatment combinations in mRC. The survival curves showed the 2-year CSS was 28.1% for no treatment group, 30.7% for only radiation group, 50.2% for only surgery group, and 66.5% for surgery plus radiation group, reaching statistical difference (*P* < 0.001) (Figure 3). It displayed that surgery of the primary cancer or radiation could improve patients’ survival and patients with combined treatments benefited the most in mRC. Additionally, the survival of patients who received surgery only was better than that of those who received only radiation, implying surgery of the primary cancer may potentially take better control of the disease. The conclusion was also verified by multivariate analysis (Table 2).

We next analyzed the characteristics differed in the four groups receiving varied treatments of the primary cancer: 1.SURG−RAD−, neither treatments; 2.SURG−RAD+, only radiation; 3.SURG+RAD−, only surgery; 4.SURG+RAD+, both treatments. The data showed that age, year of diagnosis, marital status, histological type, tumor size, *T* stage, *N* stage, *M* stage and CEA level differed in these groups, reaching statistical differences. Moreover, the percentage of patients with normal CEA level or with M1a stage in the “SURG+RAD+” group was significantly higher than that of the other three groups. Hence we selected CEA level and M stage for further study.

### Analysis of the influence of CEA level and M stage on mRC patients’ CSS based on surgery and/or radiation status

Univariate analysis demonstrated that patients with normal CEA level (*P* < 0.001) or with M1a stage had better CSS (*P* < 0.001), with statistical difference. The result of multivariate analysis was in correspondence with that of univariate analysis (Table 2).

After stratified by primary surgery status, we observed normal CEA level as well as M1a stage held a survival advantage in both surgery group and non-surgery group (*P* < 0.001, *P* < 0.001). While the survival curves showed the CSS of mRC patients was still mainly determined by the status of primary surgery regardless of CEA level or M stage (*P* < 0.001, *P* < 0.001) (Figure 4A and 4B).

Additionally, as shown in Figure 4C and 4D, we studied the influence of CEA level and M stage on the survival of mRC patients according to radiation status. The results demonstrated that normal CEA level and M1a stage still maintained their survival advantage after stratified by radiation status (*P* < 0.001, *P* < 0.001). However, unlike the analysis based on surgery status, the survival of group RAD+M1b (with radiation and with M1b stage) was worse than that of group RAD-M1a (without radiation and with M1a stage) (*P* < 0.05).

Moreover, we compared the survival differences in groups divided by both surgery and radiation status (SURG−RAD−, SURG−RAD+, SURG+RAD−, and SURG+RAD+) according to CEA level or M stage. Both univariate log-rank test and multivariate Cox regression analyses were conducted. The result implied that normal CEA level and M1a stage still predicted better prognosis after stratified by both surgery and radiation status in mRC. While the statistical significance for M1a stage was observed in all groups except for group SURG−RAD− (without surgery and without radiation), implying the survival advantage of M1a stage could not be demonstrated in the absence of treatment for primary cancer. So probably a more aggressive approach should be taken to treat the primary cancer for mRC patients with M1a stage (Table 3).

**Table 3:** Survival analysis of the influence of CEA level and M stage on mRC patients when stratified by treatment combination in the SEER database

Groups	2-year CSS	Univariate analysis		Multivariate analysis	
		Log rank χ2	*P* ^*a*^	HR (95% CI)	*P* ^*b*^
**SURG − RAD −**					
**CEA**		31.06	**< 0.001**		**0.017**
Normal	45.3%			0.623 (0.422–0.918)	
Elevated	25.6%			Reference	
***M* stage**		0.96	0.328		0.401
M1a	40.9%			0.874 (0.638–1.196)	
M1b	35.2%			Reference	
**SURG − RAD +**					
**CEA**		4.976	**0.016**		**0.053**
Normal	36.8%			0.794 (0.589–0.991)	
Elevated	30.6%			Reference	
***M* stage**		45.68	**< 0.001**		**0.006**
M1a	43.1%			0.672 (0.507–0.892)	
M1b	26.7%			Reference	
**SURG + RAD −**					
**CEA**		3.073	0.079		**0.037**
Normal	60.2%			0.778 (0.615–0.985)	
Elevated	50.3%			Reference	
***M* stage**		13.69	**< 0.001**		**0.001**
M1a	63.2%			0.648 (0.503–0.835)	
M1b	46.9%			Reference	
**SURG + RAD +**					
**CEA**		29.15	**< 0.001**		**< 0.001**
Normal	73.7%			0.596 (0.473–0.750)	
Elevated	63.2%			Reference	
***M* stage**		24.47	**< 0.001**		**< 0.001**
M1a	74.9%			0.673 (0.552–0.821)	
M1b	62.9%			Reference	

*P^a^*: *P* value for comparisons between groups by log-rank test.

*P^b^*: *P* value for comparisons between groups by multivariable Cox regression analysis adjusting for covariates.

Abbreviations: CEA, carcinoembryonic antigen; M, metastasis; mRC, metastatic rectal cancer; CSS, cancer cause-specific survival; HR, hazard ratio; CI, confidence interval; SURG−, without surgery of the primary cancer; SURG+, with surgery of the primary cancer; RAD-, without radiation; RAD+, with radiation.

Conclusively, we found the prognosis of mRC patients who underwent surgery of the primary cancer or received radiotherapy was better than that of those who didn’t, and combined treatments contributed to the best survival. Besides, normal CEA level and M1a stage predicted better prognosis for mRC patients. Hence clinicians may estimate the prognosis of mRC patients by checking their information of surgery of the primary cancer first, and then take radiotherapy, CEA level and M stage records into consideration. Overall, our results supported the addition of surgery of the primary cancer and radiation into the treatment strategy of mRC patients.

## DISCUSSION

More than half of newly diagnosed CRC in China is RC, and a considerable part of RC has already developed metastasis at diagnosis [24]. Therefore, it’s necessary to find proper therapeutic combinations and prognostic factors for mRC patients to help make better multidisciplinary treatment and follow-up plan. Hence we conducted this analysis to find potential prognostic factors for mRC patients based on the SEER database. We discovered that patients who performed surgery of the primary cancer or received radiation had better prognosis than those who didn’t in mRC. Besides, patients with normal CEA level or M1a stage maintained a survival advantage. Nevertheless, to our attention, the CSS of patients with T1 stage was similar to that of patients with T4 stage, being worse than that of patients with T2/3 stage. Moreover, we observed that patients presenting better survival were younger, more married, with smaller tumor size and with better differentiate grade, in correspondence with results of previous analyses [25–27].

For mRC patients with resectable primary cancer and resectable liver or lung metastasis, the NCCN guidelines recommend the resection of both primary and metastatic site to achieve the goal of cure. While for mRC with unresectable metastases presenting no symptoms (bleeding, obstruction and perforation), the resection of primary site is controversial. Part of studies claimed the resection of primary cancer may confer a survival benefit for mCRC patients with unresectable metastases and questioned the rational of undergoing primary surgery based solely on the presence of symptoms [16, 28–31], while other studies denied the survival differences between surgery group and non-surgery group [32, 33]. Considering the inconsistent results and the small size of these studies, as well as the existence of selection bias, it’s difficult for us to reach a consensus.

Our analysis was based on the records of the SEER database, which includes about a quarter of the whole population of the USA. In correspondence with the study based on Norwegian Rectal Cancer Registry database [34], we found the 2-year CSS was 58.7% for patients who performed primary surgery, compared with 29.3% for those who didn’t in mRC. As records in the SEER database include the code of surgical resection range, we further analyzed the influence of surgical resection range on the CSS of mRC patients. Noteworthily, it demonstrated that as long as the primary tumors were resected, the CSSs of patients would be similar regardless of varied resection ranges. In addition, the analysis between surgery group and non-surgery group showed that patients who performed the surgery tended to be younger, with smaller tumor size, with smaller metastatic burden (M1a stage) and with normal CEA level, which was in consistent with some former studies [35, 36]. Besides, in the surgery group more patients received radiotherapy compared with non-surgery group. Considering the result of the analysis of prognostic factors above, it is not hard to find that the surgery group possessed more favorable prognostic factors, which might contribute to the survival advantage to some extent.

Moreover, we found the introduction of radiotherapy into treatment strategy improved the CSS of mRC patients. A few studies have been conducted previously attempting to clarify the role of radiation in mRC. However, the results were not solid for small cohorts and varied use of chemotherapy, not to mention the conflicting results [29, 37, 38]. While our study was based on a large-scale population, making the result more reliable. Notably, the patients who received both surgery and radiation modalities showed the best CSS (66.5%), compared with “only surgery” (50.2%), “only radiation” (30.7%) and “no surgery no radiation” (28.1%) groups. Previously, Wan JF *et al.* have drawn a similar conclusion in local advanced RC patients with older age [19], and in our case the result still made sense for mRC patients of all ages on the base of a large population cohort.

In addition, our results showed that mRC patients with normal CEA level or M1a stage presented better survival in both surgery group and non-surgery group. Further, we analyzed the prognostic significance of CEA level and M stage in subgroups divided by both surgery and radiation status. The results demonstrated that normal CEA level or M1a stage still held a survival advantage in these subgroups, all reaching statistical significance except for M1a stage in “no surgery no radiation” subgroup.

CEA is an important tumor marker in CRC and the influence of CEA level on CRC has long been studied. Preoperative high CEA levels were showed to be associated with high rates of postoperative recurrence and metastass in varied stage of CRC [39–42]. So it’s recommended to monitor the CEA level routinely during follow-up [43]. In unresectable mCRC, elevated CEA levels were commonly considered to be associated with worse survival, but the conclusion is also controversial [44, 45]. Few studies focused on the effects of CEA level on RC solely. Tarantino I *et al.* and Giessen C *et al.* demonstrated that the survival of patients with elevated preoperative CEA levels is worse than that of patients with normal CEA level in non-metastatic RC [46, 47], while the study didn’t include mRC patients in analysis. Most researches studied the role of CEA in locally advanced RC with neoadjuvant treatment [48], however, few studies focused on mRC. And our study analyzed the relationship between CEA level and the prognosis of mRC patients on the base of a large population cohort. The analysis demonstrated that normal CEA level predicted better survival in mRC, and the conclusion still made sense after stratified by surgery and/or radiation status.

Metastatic disease is classified as M1a when metastases are confined to only one site/organ in mRC, and M1b stage is used for metastases to multiple distant sites/organs or peritoneum. Our analysis observed patients with M1a stage had better survival than patients with M1b stage, implying larger metastatic loads exerted bad effects on the prognosis of mRC patients. The result here identified the survival differences existed in groups with varied metastatic burden [49, 50]. The survival benefit of M1a stage maintained in subgroups with different treatments of primary cancer except for group “no surgery no radiation”, indicating the survival advantage of smaller metastatic burden could not be displayed unless some treatments of the primary cancer have been conducted. So probably a more aggressive treatment strategy should be taken to treat the primary cancer for mRC patients with M1a stage.

The impacts of T stage on the survival of local advanced RC has been learned in prior studies, stating the prognosis of patients with T1 or T2 stage is better than that of patients with T3 or T4 stage [51, 52], but analysis of the effects of T stage on mRC is quite rare. Our study noticed the CSS of patients with T2 or T3 stage was better than that of patients with T1 or T4 stage in mRC. For mRC with T1 stage, metastasis occurred despite the low *T* stage, indicating the poor biological characteristics of primary cancer, which may lead to the short survival. And for mRC with T4 stage, the deep invasion depth showed the strong invasive and penetrating ability of primary cancer cell, which probably caused the poor survival. Other clinicopathological prognostic factors we identified in the analysis included tumor size, histological type and differentiate grade, which was in consistent with former studies [53–56].

Although this is a large population-based study focusing on mRC solely, several potential limitations inevitably exist for its inherent retrospective nature. Firstly, the study lacked the information of chemotherapy and molecule-targeted treatment for the SEER database lacks corresponding records originally, causing potential confounders in the analysis. However, the bias potentially applies to both surgery and non-surgery groups as well as to both radiation and non-radiation groups. Secondly, records in the SEER database don’t include exact rectal cancer site, physical status, nutritional status, accompanied symptoms, as well as the specific metastatic status, which prevented us from making relevant analyses. Thirdly, records in the SEER database lack the evaluation of the resectability of metastases, making it impossible for us to distinguish between resectable metastases and unresectable metastases.

In conclusion, despite the potential limitations of this study, we found surgery of the primary cancer and radiotherapy could improve the CSS of mRC patients significantly, particularly when both treatments have been conducted. Hence we suggested the addition of surgery of the primary cancer and radiation into the treatment algorithms of mRC patients, especially when the patient is with a normal CEA level and/or with M1a stage. In addition, our results recommended clinicians to take CEA level, *M* stage, *T* stage, tumor size, differentiate grade, age and marital status into consideration when estimating the prognosis of mRC patients to help make the optimal treatment and follow-up plan.

## MATERIALS AND METHODS

### Patient selection in the SEER database

The SEER database covers about 26% of the population of the USA, contains 18 population-based cancer registries and provides the information on cancer statistics. We obtained the data of mRC from January 2004 to December 2013 from the SEER database by using SEER*Stat software (SEER*Stat version 8.3.2), for the record of the American Joint Committee on Cancer (AJCC) TNM stage in the database was not available until the year of 2004. Patients meeting the inclusion criteria were included in the analysis. The inclusion criteria: (1) the location of primary tumor: Rectum (the International Classification of Diseases for Oncology, 3rd Edition [ICD-O-3] site code: C20.9) ; (2) the number of primary tumor: only one primary cancer; (3) histological type: adenocarcinoma (ICD-O-3 histological type code: 8140/3, 8210/3, 8261/3, 8263/3); mucinous adenocarcinoma (8480/3) and signet ring cell carcinoma (8490/3); (4) AJCC TNM stage: stage IV; (5) pathologically confirmed diagnosis; (6) actively follow up. And patients who met the exclusion criteria were excluded from the analysis. The exclusion criteria: (1) diagnosed by death certificate or autopsy only; (2) without known survival months or cause-specific death classification.

### Data retrieved

Records of the following demographic and clinicopathological variables were retrieved from the SEER database: gender, age at diagnosis, year of diagnosis, ethnicity, marital status; histological type, grade of differentiation, *T* stage, *N* stage, *M* stage, tumor size, the status of site-specific factor 1 (CEA), surgery of the primary site, radiation, SEER cause-specific death classification and survival months.

The codes of surgery of the primary site for RC (1998–2013): 00 and 10–14, no surgery of the primary site; 20–28, local tumor excision; 30–40, partial proctectomy; 50, total proctectomy; 60, total proctocolectomy; 70, total proctectomy or proctocolectomy with an en bloc resection of other organs, including pelvic exenteration; 80 (proctectomy Not Otherwise Specified, NOS), unknown resection range; 90 (surgery NOS), was also considered as unknown resection range here.

### Statistical analysis

The SPSS software (version 21.0) was used for statistical analysis. Chi-square test was used to test qualitative data. Student’s *t*-test was used to compare quantitative data. The CSS of mRC was calculated from the date of diagnosis to the date of cancer specific death. Deaths attributed to mRC were treated as events and deaths from other reasons or survivals at the time of last follow-up were treated as censored observations. The CSS curves were plotted by Kaplan-Meier method and analyzed by log-rank test. Multivariable Cox regression analysis was used to find the risk factors for CSS. Two sided *P* < 0.05 was considered to be statistically significant.
